# Editorial: Challenges and perspectives for improved understanding and management of multifaceted co-infection

**DOI:** 10.3389/fimmu.2026.1791801

**Published:** 2026-03-18

**Authors:** Vartika Srivastava, Aijaz Ahmad

**Affiliations:** 1 Department of Cancer Sciences, Cleveland Clinic, Cleveland, OH, United States; 2 Clinical Microbiology and Infectious Diseases, Faculty of Health Sciences, School of Pathology, University of the Witwatersrand, Johannesburg, South Africa; 3 Division of Pulmonary, Allergy and Critical Care Medicine, Stanford University School of Medicine, Stanford, CA, United States

**Keywords:** host–pathogen interactions, immune dysregulation, multifaceted co-infection, pathogen–pathogen crosstalk, translational infection management

Infectious diseases rarely occur in isolation, and co-infections involving multiple pathogens are increasingly recognized as a major challenge for clinicians, researchers, and public health authorities. Multifaceted co-infection can alter disease severity, compromise diagnostic accuracy, and complicate therapeutic decision-making, yet current frameworks for understanding and managing these interactions remain fragmented. Despite growing evidence from clinical and experimental studies, significant gaps persist in how different pathogens, host immune responses, and environmental factors interact over time, especially in vulnerable populations. Therefore, advancing therapeutic strategies and improving clinical outcomes requires a detailed study of how co-infecting pathogens evolve over time, clarification of the molecular mechanisms that shape their interplay, and evaluation of their combined effects on host cellular pathways.

In this Research Topic, several studies provide novel insights into the complex immune dynamics underlying various types of co-infection. For instance, a study by Wang et al. focused on Epstein–Barr virus (EBV) infection, which is frequently observed in patients with severe fever with thrombocytopenia syndrome (SFTS). Their study aimed to characterize the impact of EBV infection on clinical outcomes and the associated immunological and inflammatory profiles in SFTS patients. The researchers found that the impact of EBV DNA positivity on mortality was especially evident in older patients, individuals admitted later in the disease course, and those with underlying comorbidities. In addition, immunological analysis of EBV-positive patients showed a substantial increase in B-cell frequency, accompanied by marked decreases in CD3+, CD4+, and CD8+ T-cell counts, with EBV DNA levels closely associated with the proportion of B cells. Furthermore, circulating IL-6 and IL-10 concentrations were significantly elevated in the EBV-positive group, and EBV DNA levels showed a strong positive correlation with IL-10.

Another contribution by Hu et al. sought to characterize the bacterial and fungal co-infecting pathogens in Severe Fever with Thrombocytopenia Syndrome (SFTS) and identify prognostic factors associated with mortality among co-infected patients. SFTS is an emerging infectious disease that is becoming a growing concern for global public health authorities. Most isolates were obtained from respiratory specimens. Among all isolated fungal pathogens, *Aspergillus fumigatus* was the most frequently detected species, followed by *Candida albicans*. Among bacterial isolates, *Klebsiella pneumoniae* and *Acinetobacter baumannii* were the predominant organisms. Patients with these co-infections experienced significantly prolonged hospitalizations, incurred higher medical expenses, and showed increased mortality. The authors concluded that bacterial/fungal co-infections are highly prevalent and clinically impactful in SFTS patients, warranting enhanced microbial surveillance and targeted intervention.

Focusing on HIV and Chronic hepatitis C virus (HCV) co-infection, Psomas et al. investigated the changes in bone mineral density and bone quality parameters after successful HCV treatment with direct-acting antivirals (DAA) that differed from a matched control population without HIV or HCV. The authors also sought to understand how DAA impacted inflammatory markers, especially in people with HIV and HCV co-infection. HCV cure with DAA was associated with decreased systemic inflammation without changes in bone parameters. Whereas a significant increase in bone formation markers was observed in HIV/HCV co-infected individuals.

In another study, Zhao et al. have investigated co-infection of human immunodeficiency virus (HIV) and *Mycobacterium tuberculosis* (Mtb) in AIDS patients. The authors applied single-cell RNA sequencing (scRNA-seq) to peripheral blood mononuclear cells from healthy controls, and from participants newly diagnosed with HIV mono-infection or HIV-Mtb co-infection, before therapy initiation. Their study provides a single-cell roadmap of HIV-Mtb co-infection and identifies Th1/Th17 imbalance and reconfiguration of MHC-I-biased T-cell signaling as candidate targets for restoring immune homeostasis.

Fan et al. conducted a study aimed at systematically analyzing the clinical characteristics, pathogen spectrum, and independent risk/protective factors for coinfection in Severe Fever with Thrombocytopenia Syndrome (SFTS) patients, and to explore optimal therapeutic regimens to reduce the fatality rate in SFTS patients with coinfection. The study found a high proportion of SFTS patients with coinfection (bacterial and fungal), which significantly worsens their clinical prognosis.

In another contribution, Meyer et al. investigated antibody responses in individuals infected with onchocerciasis following multiple doses of different COVID-19 vaccines, comparing mRNA- and vector-based platforms, accounting for co-infections, and contrasting these responses with those observed in healthy endemic controls. The study concluded that COVID-19 vaccines elicit robust humoral immune responses despite underlying onchocerciasis infection. However, parasitic status and microfilarial burden may attenuate vaccine-induced responses, underscoring the complexity of evaluating vaccine efficacy in settings with uncertain infection histories, co-infections, and polyparasitism common to helminth-endemic regions. These findings highlight the need for further investigation into parasite-driven immunomodulatory mechanisms that may influence COVID-19 vaccine effectiveness and inform future vaccine development.

Ahmad et al. worked towards understanding severe complications of Influenza-associated pulmonary aspergillosis (IAPA), which is associated with higher mortality compared to influenza infection alone. The authors investigated the role of IL-17 and Type 17 immune signaling components during IAPA. In the study, gene and protein expression levels showed that IL-17 and Type 17 immune cytokines and antimicrobial peptides were downregulated during IAPA compared with those in mice infected solely with *A. fumigatus*. Furthermore, the study highlights the need for future research to elucidate the immune mechanisms that increase susceptibility to fungal infections.

Another contribution by Bohórquez et al. explores human immunodeficiency virus (HIV) co-infection with tuberculosis (TB), caused by Mycobacterium tuberculosis (*Mtb*), and describes the development of a novel humanized mouse model. The authors demonstrated that humanized NSG-SGM3 mice faithfully recapitulate the pathological, immunological, and metabolic features of HIV and *Mtb* infections, as well as their co-infection, establishing this model as a reproducible and robust small-animal system for studying HIV/*Mtb* co-infection.

In conclusion, the studies presented in this Research Topic highlight the complexity and clinical significance of co-infections, particularly in immuno-compromised populations. Collectively, these contributions elucidate the multifaceted interplay between host immunity and pathogenic organisms. As our understanding of co-infection biology continues to advance, it is increasingly evident that effective strategies for diagnosis, treatment, and prevention must adopt an integrated approach that considers both pathogen dynamics and host-specific factors.

